# Impact of aquaculture on distribution of dissolved organic matter in coastal Jeju Island, Korea, based on absorption and fluorescence spectroscopy

**DOI:** 10.1007/s11356-021-15553-3

**Published:** 2021-07-31

**Authors:** Jeonghyun Kim, Yeseul Kim, Sung Eun Park, Tae-Hoon Kim, Bong-Guk Kim, Dong-Jin Kang, TaeKeun Rho

**Affiliations:** 1 Department of Earth and Marine Sciences, College of Ocean Sciences, Jeju National University, Jeju, 63243 Republic of Korea; 2 Marine Environmental Research Center, Korea Institute of Ocean Science and Technology (KIOST), Busan, 49111 Republic of Korea; 3 Department of Ocean Science, University of Science and Technology (UST), Daejeon, 34113 Republic of Korea; 4 Marine Environment Research Division, National Institute of Fisheries Science (NIFS), Busan, 46083 Republic of Korea; 5 Department of Oceanography, Faculty of Earth Systems and Environmental Sciences, Chonnam National University, Gwangju, 61186 Republic of Korea; 6 Underwater Survey Technology 21, Incheon, 21999 Republic of Korea; 7 Instrumental Development and Management Center, Korea Institute of Ocean Science and Technology (KIOST), Busan, 49111 Republic of Korea

**Keywords:** Aquaculture, Anthropogenic organic pollution, Chromophoric dissolved organic matter, Fluorescence excitation-emission matrix (EEM) spectroscopy, PARAFAC, Dissolved organic matter

## Abstract

In Jeju Island, multiple land-based aquafarms were fully operational along most coastal region. However, the effect of effluent on distribution and behaviours of dissolved organic matter (DOM) in the coastal water are still unknown. To decipher characteristics of organic pollution, we compared physicochemical parameters with spectral optical properties near the coastal aquafarms in Jeju Island. Absorption spectra were measured to calculate the absorption coefficient, spectral slope coefficient, and specific UV absorbance. Fluorescent DOM was analysed using fluorescence spectroscopy coupled with parallel factor analysis. Dissolved organic carbon (DOC) and total dissolved nitrogen (TDN) were measured using high-temperature catalytic oxidation. The DOC concentration near the discharge outlet was twice higher than that in natural groundwater, and the TDN concentration exponentially increased close to the outlet. These distribution patterns indicate that aquafarms are a significant source of DOM. Herein, principal component analysis was applied to categorise the DOM origins. There were two distinct groups, namely, aquaculture activity for TDN with humic-like and high molecular weights DOM (PC1: 48.1%) and natural biological activity in the coastal water for DOC enrichment and protein-like DOM (PC2: 18.8%). We conclude that the aquafarms significantly discharge organic nitrogen pollutants and provoke in situ production of organic carbon. Furthermore, these findings indicate the potential of optical techniques for the efficient monitoring of anthropogenic organic pollutants from aquafarms worldwide.

## Introduction

Over the last few decades, there has been a rapid development in global aquaculture industries with the aim of providing adequate food resources for the exponentially growing population in Asia and the Pacific region (FAO 2018). This accelerated development of the aquaculture industry has resulted in threats to the coastal environment with the increase in the discharge of organic waste and inorganic nutrients into coastal aquatic environments. Although many countries have established environmental standards for sustainable aquaculture operations, the triggering of severe pollution is still a major concern for improperly managed and overcrowded facilities (Qin et al. 2005; Strain and Hargrave 2005). In previous studies, the direct excretion, by-products of microbial metabolic activity, and excessive feeding in aquafarms have contributed to the presence of highly enriched organic and inorganic substances in effluent (Brinker et al. 2005; Green et al. 2002; Sindilariu 2007). This has directly led to negative environmental impacts, such as eutrophication, water quality deterioration, and red tide downstream of the discharge outlet (Rosa et al. 2013). The aquaculture-driven eutrophication and environmental problems have been reported worldwide and are becoming increasingly frequent (Zhang et al. 2019).

**Fig. 1:**
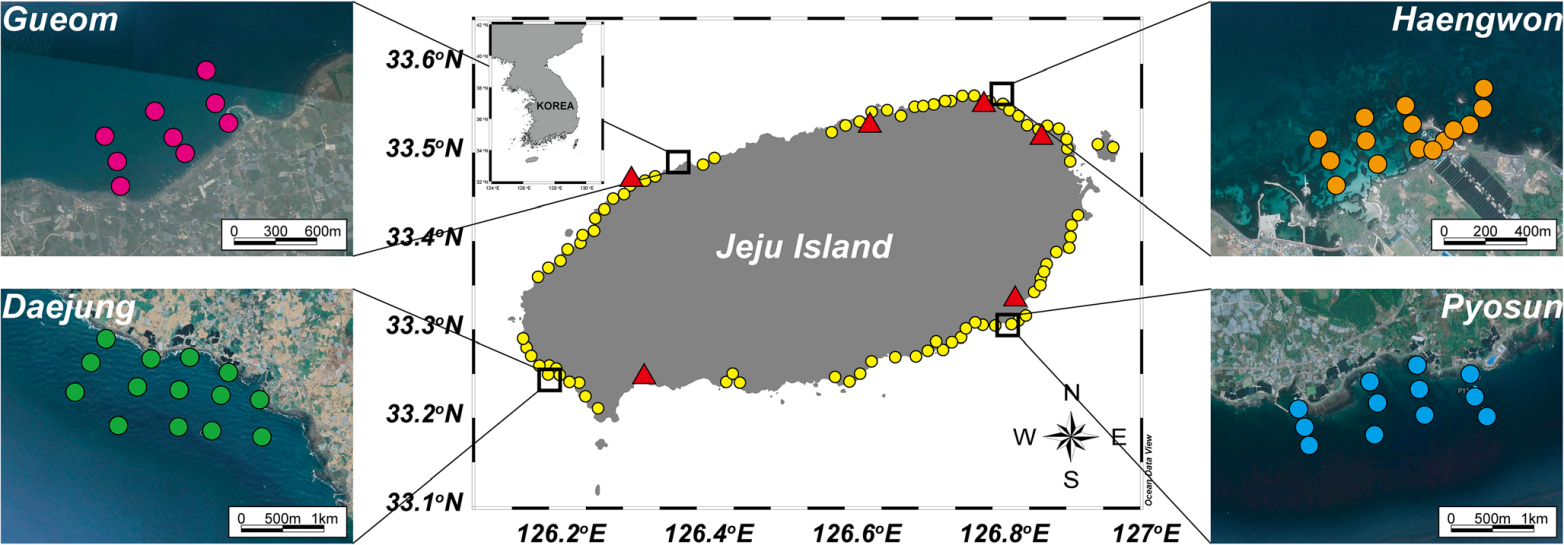
Study regions and sampling stations around Jeju Island, Korea. The yellow circles indicate the location of coastal aquafarms. The orange, green, and blue circles were the sampling stations near the aquafarms in area Haengwon, Daejung, and Pyosun districts, respectively. The red circles represent the sampling stations in area Gueom, as the control group, which are located in more than 5 km distance from the aquafarms. The red triangles represent the study sites for physicochemical parameters of the coastal groundwater in previous studies (Cho et al. 2021; Kim and Kim 2017a; Kim and Kim 2017b; Kim et al. 2013; Song et al. 2018)

Although Jeju island is on the branch of the oligotrophic Kuroshio Current, environmental problems associated with eutrophication have occurred in the coastal region, eventually leading to economic damage. Significant macroalgal blooms and green tide outbreak have been since the early 2000s. Approximately 10,000 tons of macroalgae has produced annually in the nearshore of Jeju Island (http://hei.jeju.go.kr). Samanta et al. (2019) demonstrated that the aquaculture effluent contributes significantly to coastal nutrient budgets and eventually Ulva blooms using algal isotope signatures (δ^13^C, δ^15^N, and δ^18^O). In addition, mass mortality events of farmed fishes have occurred in the coastal aquafarms, and it seems be associated with a decline in water quality. Approximately 360 aquafarms are fully operational in most coastal areas (Fig. 1). Bastard halibut (*Paralichthys olivaceus*) is a dominant fish, accounting for 95% of all fish grown in land-based aquaculture facilities with flow-through systems. These culture facilities use a mixture of offshore seawater (from approximately 500 m offshore) and groundwater (from up to 100 m underground) to support conditions favourable for the rearing of the best-quality halibut, because of organic carbon-depleted groundwater with consistent hydrological properties (17–18 °C and pH 7.2) throughout the year. The effect of the effluent on the water quality in the coastal environment needs to understand.

Traditionally, to evaluate the water quality of aquaculture-driven bulk wastewater, chemical oxygen demand, biological oxygen demand, and total organic carbon (TOC) were measured. Recently, an ultra-high resolution mass spectrometry (UHR-MS) technique such as Fourier transform ion cyclotron resonance mass spectrometry (FTICR-MS) has enabled the investigation of the molecular composition of organic pollutants (Kamjunke et al. 2017). However, these chemical analysis techniques are costly and time-consuming. The optically sensitive fractions of DOM, which are characterised by light absorption and fluorescence, are termed chromophoric-DOM (CDOM) and fluorescent-DOM (FDOM), respectively. The spectral properties of CDOM and FDOM have been used to determine quantitative (concentration of organic matter and pollutants) and qualitative information (origins and molecular weight) simultaneously (Stedmon and Nelson 2015). In addition, optical analysis techniques have the advantage of being relatively fast (< 30 min) and low-cost methods. Thus, CDOM and FDOM can be used to characterise DOM, and large spatial scales of water quality monitoring are possible. The detection of absorbance provides ultraviolet (UV) and visible (VIS) absorption spectra, whose properties depend on the concentration and molecular structure of the chromophore in DOM (Stedmon et al. 2000). In addition, a series of excitation and emission fluorescence signals across the broad UV–VIS spectra visually demonstrated three-dimensional excitation-emission matrix spectroscopy (EEMs). Using parallel factor analysis (PARAFAC), the spectral pattern and optical intensity of fluorescent EEMs were used to decipher the DOM characteristics in aquaculture. Nimptsch et al. (2015) revealed that large amounts of anthropogenic dissolved organic carbon (DOC) with the most biodegradable protein-like FDOM derived from land-based aquaculture facilities are being discharged into the river system in the North Patagonian region. Hambly et al. (2015) identified the origin of organic matter in recirculating aquaculture systems (RASs) as feed, tap water, and processes associated with fish farming and water purification using EEMs-PARAFAC. Yamin et al. (2017) observed the significant accumulation of humic-like organic matter in the culture water during operation of the zero-discharge RAS using EEMs-PARAFAC.

This study aimed to determine the core factors contributing to the behaviours and origins of DOM near aquafarms and influence of aquaculture-driven effluents in the pristine coastal Jeju Island using optical properties. We focused on the application of multiple optical properties of CDOM and FDOM (e.g. absorption and fluorescence) coupled with statistical analysis (including PARAFAC and principal component analysis (PCA)) for tracing anthropogenic DOM with carbon and nitrogen contents. The approach was based on the hypothesis that optical analysis, which is cost- and time-efficient, can be applied to distinguish between the anthropogenic and natural origins of DOM. The results of this study indicate that optical analysis has potential as an efficient monitoring method for anthropogenic organic pollutants from aquafarms worldwide.

## Materials and methods

### Sampling campaigns

Sampling campaigns were conducted near three major coastal aquafarms (Haengwon, Daejung, and Pyosun districts), where multiple aquaculture facilities are concentrated (Fig. 1). In addition, we performed sampling in one normal coastal region (Gueom), designated as the control group, which is located more than 5 km away from the coastal aquafarms (Fig. 1). Sampling campaigns were conducted in April, July, and September 2018, to determine the seasonal effects on water quality with different hydrological properties. At these sampling sites, we considered three types of samples, namely, drainage samples, coastal water samples near aquafarms, and offshore samples. Drainage samples were directly obtained at the discharge outlet in the aquafarm region. The coastal water samples near the aquafarms were obtained from within 1.5 km from the outlet (Fig. 1), and sampling was performed on sit-in-kayaks because of the extremely shallow depths (avg. 2 m). We considered coastal water samples at 9–15 sampling stations with distance from the outlet to demonstrate the spread range of the drainage. Water sampling was conducted using an acid-clean Nalgene HDPE plastic beaker (Thermo Fisher Scientific, MA, USA).

### Analysis of physicochemical parameters

Temperature and salinity were measured using a portable CastAway-CTD sensor (YSI Inc., OH, USA). The pH values were measured using a portable probe (YSI Pro1030). These sensors were calibrated using the YSI standard solutions (YSI3169 Conductivity Calibrator for salinity and pH buffer solutions (YSI3821, 3822, and 3823) for pH) before each sampling campaign.

Samples for DOC and total dissolved nitrogen (TDN) were vacuum filtered through pre-combusted Whatman GF/F glass fibre filters (500 °C for 4 h; pore size: 0.7 μm; Whatman Inc., NJ, USA). To prevent microbial degradation, the filtrate was acidified to a pH of 2 using 6 M hydrochloric acid (Sigma-Aldrich, Germany) and placed in pre-combusted (500 °C for 4 h) EPA glass amber vials (Fisher Scientific, NH, USA). The DOC concentration was measured via high-temperature catalytic oxidation using a TOC analyser (TOC-L, Shimadzu, Japan). The TDN was analysed simultaneously with DOC using the same TOC analyser equipped with a total nitrogen unit (TNM-L). To achieve high accuracy, the system blank was reduced until the signal from the DOC-free distilled water was consistent within the limit of detection (< 0.1 mg/L for DOC, < 0.1 mg/L for TDN). The accuracy of the DOC and TDN concentrations was verified with every sample run using deep-sea references (DSR: 0.49–0.53 mg/L for DOC and 0.43–0.46 mg/L for TDN, University of Miami). Our DSR measurement results were found to be in good agreement with the consensus values (within 2%).

### Analysis of CDOM and FDOM

An aliquot of the filtrate was re-filtered through pre-rinsed 0.2 μm polycarbonate filters (Nuclepore™ Track-Etched Membranes, Whatman Inc., NJ, USA) to remove bacterial cells, and stored in pre-combusted EPA amber glass vials in a refrigerator below 4 °C.

UV–VIS absorbance spectra and fluorescence EEMs were measured simultaneously using a spectrofluorometer (Aqualog, HORIBA Jobin Yvon, NJ, USA) within one week of filtration. CDOM absorbance was blank-corrected, and a baseline correction was applied at 600 nm, assuming negligible CDOM absorption at that wavelength. The CDOM absorbance was further converted into the Napierian absorption coefficient [*a*_*CDOM*_(λ)], obtained from the following equation:

1 $$ {a}_{CDOM}\left(\lambda \right)=\frac{2.303\times A\left(\lambda \right)}{L} $$

where *A*(λ) denotes the absorbance at a specific wavelength (m^-1^) and *L* denotes the cuvette path length in metres (Eq. 1). The term *a*_*CDOM*_(λ) is generally adopted as a proxy to assess the CDOM content in a water sample. The spectral slope coefficient for the interval of 250–600 nm (*S*_*250–600*_, nm^-1^) was derived from the CDOM absorption spectra by fitting the absorption spectra to an exponential decay equation:

2 $$ {a}_{\lambda }={a}_{\lambda_{ref}}{e}^{-S\left(\lambda -{\lambda}_{ref}\right)} $$

where *a* denotes the Napierian absorption coefficient (m^-1^), λ denotes the wavelength (nm), and λ_ref_ denotes the reference wavelength (nm) (Eq. 2). The specific UV absorbance (*SUVA*_*254*_, unit: L mgC^-1^ m^-1^) was derived from the UV absorbance at 254 nm normalised to the DOC concentration.

The analysis was performed using scanning emission wavelengths of 249.1–599.2 nm in 4.5-nm increments and excitation wavelengths of 251–600 nm in 3-nm increments with an integration time of 5 s (Kim et al. 2020). The Rayleigh and Raman scattering peaks of the EEMs were replaced with missing values. The inner filter effect was further corrected with the UV absorbance values of each sample using the Aqualog Software. The non-negativity constraint was applied to all three modes. PARAFAC modelling of 423 EEM data was performed using the Solo+MIA software package (Eigenvector Research Inc., WA, USA), and two data points were removed as outliers. A core consistency test was applied to validate the model and identify the appropriate number of PARAFAC components (Bro and Kiers 2003). The fluorescence intensities of the samples were normalised daily to the area under the Raman peak of Milli-Q water at an excitation wavelength of 350 nm; these values were expressed as Raman Units (R.U.) (Lawaetz and Stedmon 2009).

### Statistical analysis

For the comparison between variables, analysis of variance (ANOVA) was conducted using Microsoft Excel 2016 (Microsoft, WA, USA). The reported values of the measurements were expressed as the average and standard deviation using Microsoft Excel 2016. The smooth trends of each parameter with distance from the discharge outlet of aquafarms were plotted based on the Loess function using the ggplot2 package (version 3.2.1) for R and RStudio (version 1.2.5033, MA, USA).

## Results

### Physicochemical parameters

The water temperature of the coastal water revealed the typical trend of monsoon regions (24.19 ± 2.36 °C in July and August 2018, 16.01 ± 0.47 °C in April 2018); however, salinity and pH demonstrated no seasonal pattern. The salinity and pH values were statistically distinct for the drainage (32.03 ± 3.67 and 7.96 ± 0.20), the coastal water near the aquafarm outlet (33.16 ± 0.98, 8.16 ± 0.11), and the normal coastal water as the control site (33.67 ± 0.69 and 8.23 ± 0.05) (ANOVA, *p* < 0.005; Figure S1, Table S1).

**Fig. 2:**
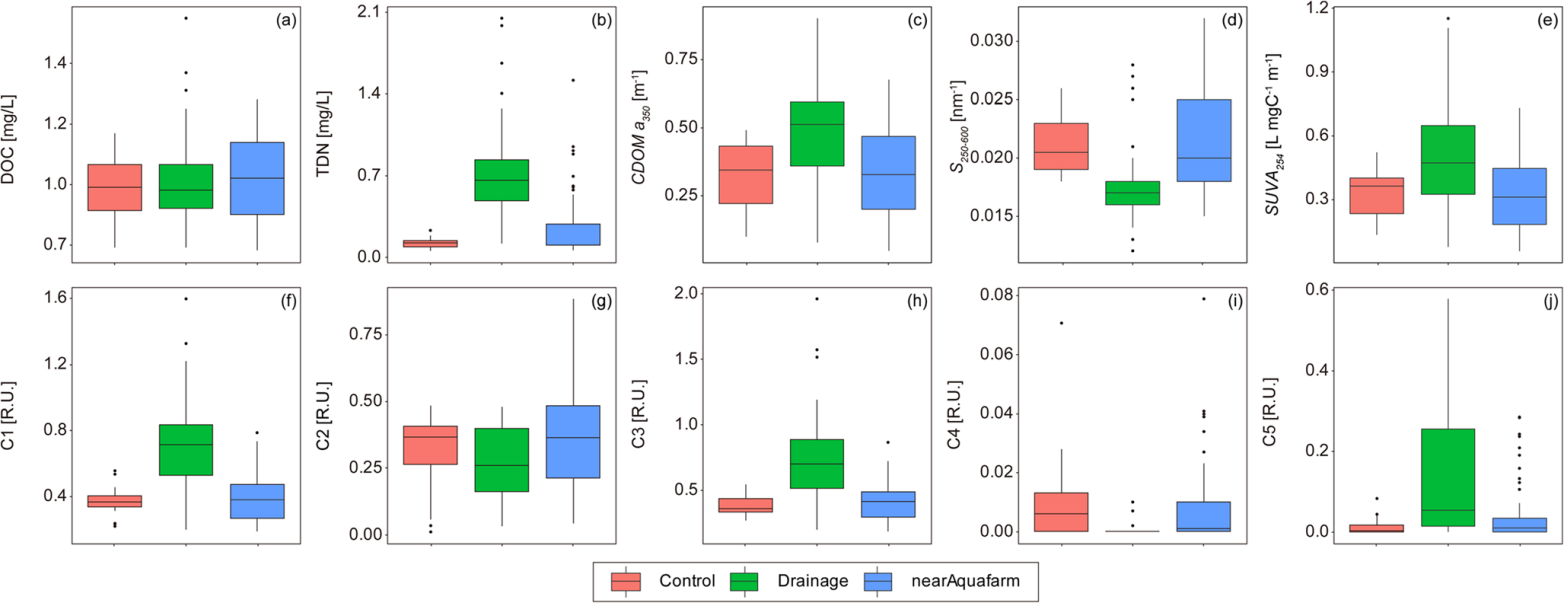
The comparison of the DOC (a), TDN (b), absorption coefficient (c), spectral slope (d), specific UV absorbance (e), and fluorescence intensities of each component (f – j) between the control site (red), the drainage (green), and near-coastal water from the aquafarm outlet (blue)

The DOC concentrations were similar for the three different sample groups: 0.95 ± 0.15 mg/L for the control site, 0.98 ± 0.20 mg/L for the drainage samples, and 0.99 ± 0.17 mg/L for the coastal water near the aquafarm outlet (ANOVA, p = 0.58; Figure 2(a), Table S1). However, the DOC concentration for the drainage samples was approximately two times higher than that of the DOC-depleted groundwater on Jeju Island, as reported in previous studies (0.31 ± 0.13 mg/L in Kim and Kim (2017a), 0.25–0.67 mg/L in Kim et al. (2013)). The DOC concentrations of groundwater were significantly lower than those in the deep northern Pacific Ocean (0.41 ± 0.01 mg/L in Hansell and Carlson (1998)). The TDN concentrations were highest in the drainage samples (0.74 ± 0.46 mg/L), followed by that in the coastal water near the aquafarm outlet (0.25 ± 0.26 mg/L) and the control site (0.12 ± 0.05 mg/L) (ANOVA, *p* < 0.001; Fig. 2(b), Table S1). The TDN concentration in the control site was similar to the endmember values in the inner (0.12 mg/L) and middle (0.17 mg/L) parts of Kahana Bay, Hawaii (Garrison et al. 2003).

### Characteristics of CDOM

The coefficient of CDOM *a*_*350*_ was higher for the drainage samples (0.47 ± 0.21 m^-1^) than for the control site (0.32 ± 0.13 m^-1^) and the coastal water near the aquafarm outlet (0.32 ± 0.16 m^-1^) (ANOVA, *p* < 0.001; Figure 2(c), Table S1). The value of *S*_*250–600*_ for the drainage samples was 0.018 ± 0.004 nm^-1^, increasing to 0.022 ± 0.004 nm^-1^ for the coastal water and 0.021 ± 0.003 nm^-1^ for the control site (ANOVA, p < 0.001; Figure 2(d), Table S1). The value of *SUVA*_*254*_ was higher for the drainage samples (0.49 ± 0.25 L mg C^-1^ m^-1^) than those for the control site (0.32 ± 0.12 L mg C^-1^ m^-1^) and the coastal water near the aquafarm outlet (0.32 ± 0.16 L mg C^-1^ m^-1^) (ANOVA, *p* < 0.001; Fig. 2(e), Table S1).

### Characteristics of FDOM using the PARAFAC model

**Fig. 3:**
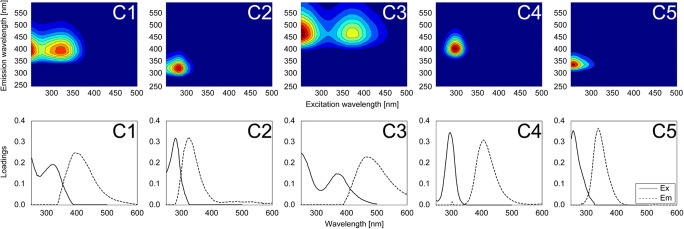
Excitation-emission matrix spectroscopy contour plots (upper row) and loadings (lower row) of five components (C1–C5) determined by the parallel factor analysis model. The solid and dotted lines represent the excitation and emission wavelengths, respectively

One to six components were retained in the PARAFAC model (Fig. 3). Five components (C1–C5) were identified by correlation with the fluorescence spectra of components in previous studies from the OpenFluor database with Tucker congruence coefficients exceeding 0.95 (Murphy et al. 2014). For the five-component model, the explained variance was 97.452% for the dataset and the core consistency was 48%. The FDOM components were distinguished by three humic-like (C1, C3, and C4) and two protein-like (C2 and C5) components according to each peak location and the literature (Table 1, Fig. 3).

**Table 1:** The optical properties of FDOM components identified by the PARAFAC model from the coastal Jeju Island. Component matches (> 0.95 Tucker congruence coefficient) were identified in previous studies from the OpenFluor database (Murphy et al. 2014).

Component	Max. wavelength (Ex/Em, unit: nm)	Description	Number of matches	Previous studies
1	251(320) / 394	UV humic-like Wastewater, anthropogenic Possible microbial reprocessing	33	C2 (Hambly et al. 2015) C1 (Heibati et al. 2017) C3 (Murphy et al. 2011)
2	281 / 326	Protein-like, amino acid-like Ocean productivity	24	C5 (Hambly et al. 2015) C4 (Osburn et al. 2016) C5 (Shutova et al. 2014)
3	251(365) / 464	Terrestrial humic-like Recalcitrant UV humic-like	30	C4 (Cohen et al. 2014) C2 (Kulkarni et al. 2018) C2 (Shutova et al. 2014)
4	296 / 408	Marine humic-like Microbial production	5	C6 (Walker et al. 2009) C5 (Kowalczuk et al. 2013) C2 (Wünsch et al. 2015)
5	257 / 339	Protein-like, Tryptophan-like Biological production Freshly production	4	C3 (Nimptsch et al. 2015) C4 (Dainard et al. 2015) C4 (Wünsch et al. 2015)

C1 (Max_ex/em_ = 251(320)/394 nm) resembles wastewater organic matter and/or microbial-origin organic matter from terrestrial and marine sources (Hambly et al. 2015; Heibati et al. 2017; Murphy et al. 2011; Stedmon and Markager 2005). C2 (Max_ex/em_ = 281/326 nm) is similar to the protein-like component (Hambly et al. 2015; Osburn et al. 2016; Shutova et al. 2014). C3 (Max_ex/em_ = 251(365)/464 nm) corresponds to FDOM_H_, traditionally documented to originate from terrestrial organic matter (Cohen et al. 2014; Kulkarni et al. 2019; Shutova et al. 2014). C4 and C5 have extremely limited matches with respect to the OpenFluor database. C4 (Max_ex/em_ = 296/408 nm) matched components from five models in the OpenFluor database (Kowalczuk et al. 2013; Walker et al. 2009; Wünsch et al. 2015), and the location of the maximum peak was similar to that of the marine humic-like component of the recognised M peak (Coble 2007). However, the shape of C4 was too sharp because the humic component typically exhibited a broad wavelength peak. In addition, C5 (Max_ex/em_ = 257/339 nm) matched only four models in the OpenFluor database (Dainard et al. 2015; Nimptsch et al. 2015; Wünsch et al. 2015) and its location was similar to that commonly reported as a protein-like component associated with recent biological production.

The fluorescence intensities of C1, C3, and C5 were significantly higher in the drainage samples (0.707 ± 0.316 R.U., 0.731 ± 0.400 R.U., and 0.143 ± 0.163 R.U., respectively) than in the control site (0.365 ± 0.088 R.U., 0.377 ± 0.083 R.U., and 0.014 ± 0.022 R.U., respectively) and coastal water near the aquafarm outlet (0.392 ± 0.138 R.U., 0.414 ± 0.139 R.U., and 0.039 ± 0.070 R.U., respectively; Figures 2(f,h,j), Table S1). In contrast, the fluorescence intensities of C2 and C4 were significantly lower in the drainage samples (0.261 ± 0.141 R.U. and 0.001 ± 0.002 R.U., respectively) than in the control site (0.302 ± 0.146 R.U. and 0.007 ± 0.008 R.U., respectively) and coastal water near the aquafarm outlet (0.354 ± 0.167 R.U. and 0.008 ± 0.013 R.U., respectively; Figs. 2(g,i), Table S1).

### Principal component analysis

**Fig. 4:**
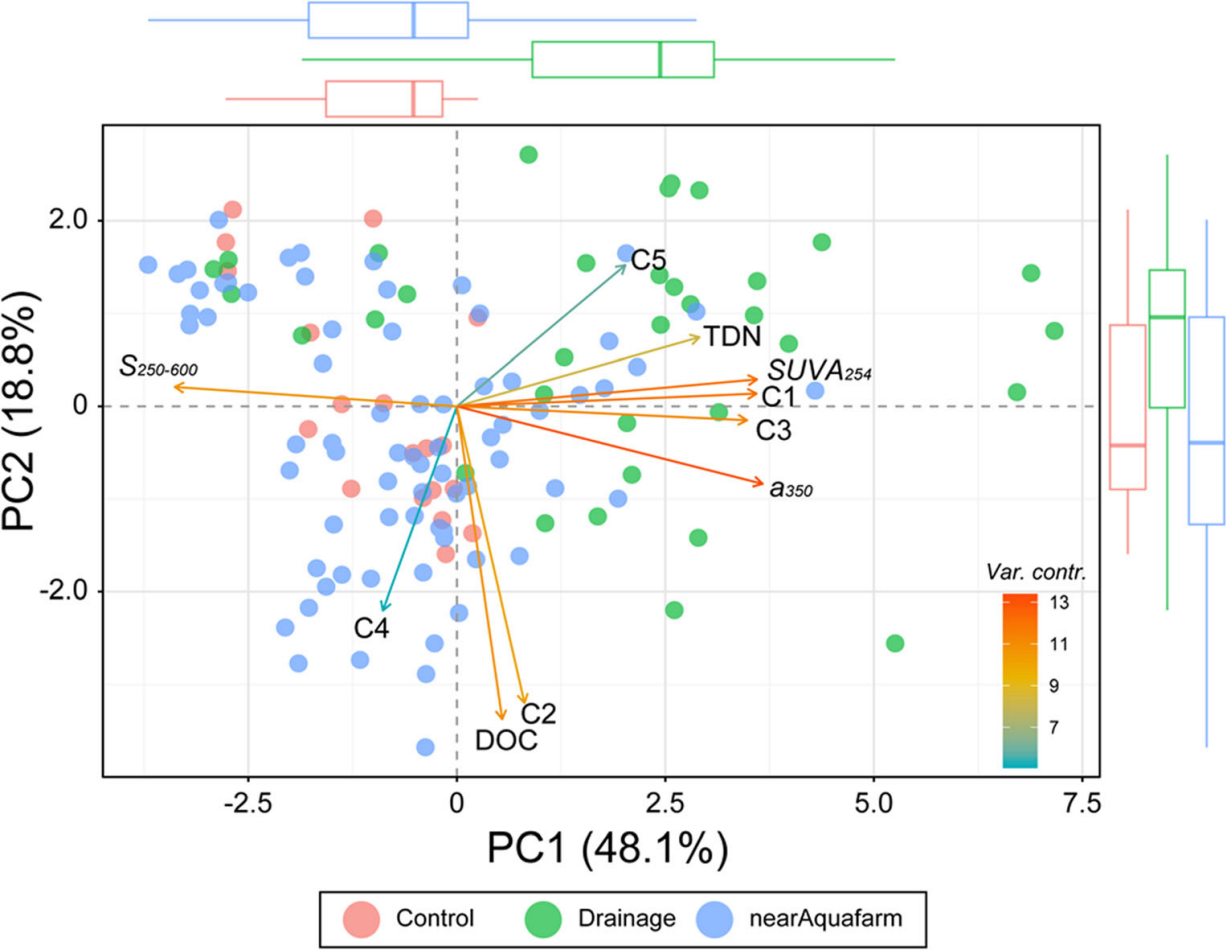
The PCA biplot of scores and loadings for the dataset of the control site (red), drainage (green), and near-coastal water from the aquafarm outlet (blue). Loadings for the DOM parameters (DOC, TDN, *a*_*350*_, *S*_*250-600*_, *SUVA*_*254*_, and the PARAFAC components) are indicated as vector arrows, and the colour of the arrows shows variable contributions to the principal axes. The boxplots at the up and right sides indicate the variation of PC1 and PC2, respectively

PCA was conducted to visualise the distribution behaviour and origins of DOM from aquaculture facilities (Fig. 4). The PCA model included the DOC, TDN, *a*_*350*_, *S*_*250–600*_, *SUVA*_*254*_, and FDOM components (C1–C5). The first and second principal components of PCA (PC1 and PC2) accounted for 48.1% and 18.8% of the variance in the dataset, respectively. Loading plots of the PCA results clearly revealed two different groups of DOM parameters. Along PC1, the loadings of TDN, *a*_*350*_, *SUVA*_*254*_, C1, and C3 were clustered and positive, whereas those of *S*_*250–600*_ were negative. In this study, the scores of the drainage group were mostly positive for PC1, whereas those of the control site and coastal water near the aquafarm outlet were relatively negative. In contrast, DOC and C2 were clustered and showed negative loadings along PC2. The variable contributions of C4 and C5 were relatively lower than those of the other parameters.

## Discussion

### Physicochemical characteristics of aquaculture effluent

The relatively lower values of pH and salinity in the coastal region near the coastal aquafarms in comparison with those in the control sites indicate the slight influence of the fresh groundwater mixture (Figure S1). Because the groundwater in Jeju Island exhibits typically consistent hydrological properties (temperature of 17–18 °C and a pH of ~ 7.2) throughout the year, the mixture of groundwater pumped from underground with offshore seawater has been used as culturing water. Lee and Kim (2015) reported that the pH value of fresh groundwater was approximately 7.5 and sharply increased up to 10 in the subterranean estuary of Jeju Island due to the adsorption of protons “protonation.” Although recirculated saline groundwater is also significant in some regions, we observed relatively lower pH (7.96±0.20) and salinity (32.03±3.67) in the effluent compared to the offshore region (8.23±0.05, 33.68±0.71), indicating the mixing of the fresh groundwater rather than saline groundwater. The coastal groundwater seeps through the seabed of most coastal Jeju Island (Kim et al. 2003). Thus, the pH and salinity values at the control site also reflect the inherent physicochemical properties of groundwater. Hence, the relatively lower pH and salinity in the coastal region near the aquafarms indicate that the coastal aquafarms pump groundwater, thereby affecting the physicochemical parameters in the coastal Jeju Island. Seasonal variations in the quality of the culture water can be determined by changes in the physicochemical parameters (Benner and Opsahl 2001). This can be demonstrated by the relationship between DOM and the physicochemical properties. However, the measured DOM parameters reveal no correlation with salinity and pH in the three sampling campaigns, indicating that DOM in the wastewater is affected by random aquaculture activities rather than environmental and hydrological variations in the coastal Jeju Island.

**Fig. 5:**
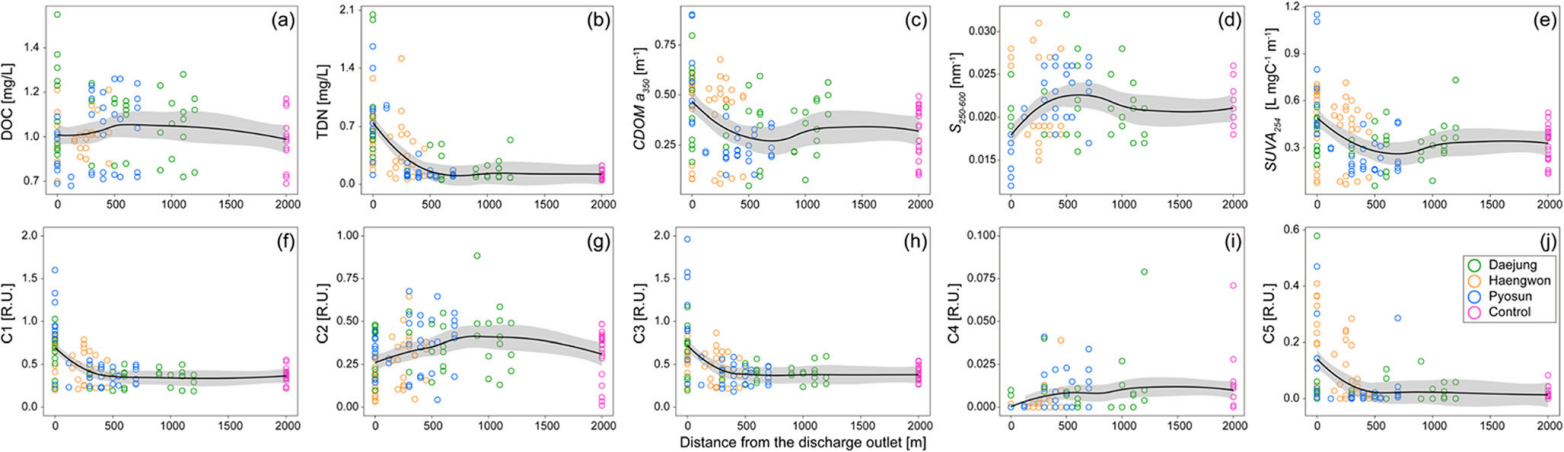
The DOC (a), TDN (b), absorption coefficient (c), spectral slope (d), specific UV absorbance (e), and fluorescence intensities of each component (f – j) with distance from the aquafarm outlet. The solid lines of each parameter indicate the spatial trend with distance from the discharge outlet based on the Loess function

To evaluate the quality of the aquaculture-driven bulk wastewater, we measured the DOC and TDN as the organic matter content, that is, carbon and nitrogen. The distribution patterns of DOC and TDN showed no significant deviation between the sampling sites (Fig. 5), indicating that the contribution of the organic pollutants from the aquafarms was similar in most coastal Jeju Island. In addition, seasonal variations in the DOC and TDN concentrations were not observed, likely due to random activities. The distribution of DOC with a distance from the outlet demonstrated significant scattering and inconclusive patterns (Fig. 5(a)). Although some data points in the drainage samples exhibited significantly high concentrations (up to 1.62 mg/L), most measurements were relatively lower than those near the coastal sea. However, the average concentrations in the drainage (0.98 ± 0.20 mg/L) were relatively higher than in the coastal groundwater in Jeju Island. Although we could not directly obtain the water intake sample from the groundwater well owing to the lack of access authority, the DOC concentration in the fresh groundwater was consistently and significantly low in the coastal Jeju Island in different seasons (Cho et al. 2021; Kim and Kim 2017a; Kim and Kim 2017b; Kim et al. 2013; Song et al. 2018). Additionally, the contribution of the fresh groundwater origin to the drainage and coastal water samples appeared to be significant based on the statistically lower salinity and pH values. Thus, we speculated that the elevated DOC concentration in the drainage samples may be attributed to the anthropogenic source (i.e. fish farming activity), because the intake of offshore water for the culture water causes a concentration increase and many DOC sources exist during aquaculture activities such as feed and faeces (Hambly et al. 2015).

Nevertheless, herein, the relatively constant level of DOC near the coastal region may be attributed to the fact that the coastal groundwater of Jeju Island is extremely pristine and serves as a major external variable contributing to lower DOC levels (Kim and Kim 2017a; Kim et al. 2013). In addition, land-based aquafarms use the mixture of groundwater and offshore water, and most facilities are carried out using flow-through systems with a high-water exchange rate were likely to affect to be less variation of DOC concentration from each sample. In contrast, relatively higher concentrations of DOC were distributed within 500–1000 m from the discharge outlet (Fig. 5(a)). This pattern did not conform to the results of Nimptsch et al. (2015), who reported a significant increase in DOC concentrations close to the outlet.

The TDN concentrations exponentially increased when close to the aquafarm outlet (Fig. 5(b)), indicating that the wastewater of the aquafarms contained excess nitrogen sources and contributed significantly to the TDN budget in the coastal region. Notable enrichment of TDN was observed within 500 m from the outlet, suggesting a direct impact of the discharge of organic waste and dilution in this area. The primary nitrogen sources from aquacultures may be faeces, unconsumed feed, and metabolic by-products in inorganic and organic forms (Wang et al. 2012). In addition, the relatively elevated TDN level in the coastal water near the aquafarms in comparison with that in the control samples (Fig. 5(b)) could be attributed to the input of wastewater.

### Factors affecting characteristics of DOM using PCA coupled with optical properties

**Fig. 6:**
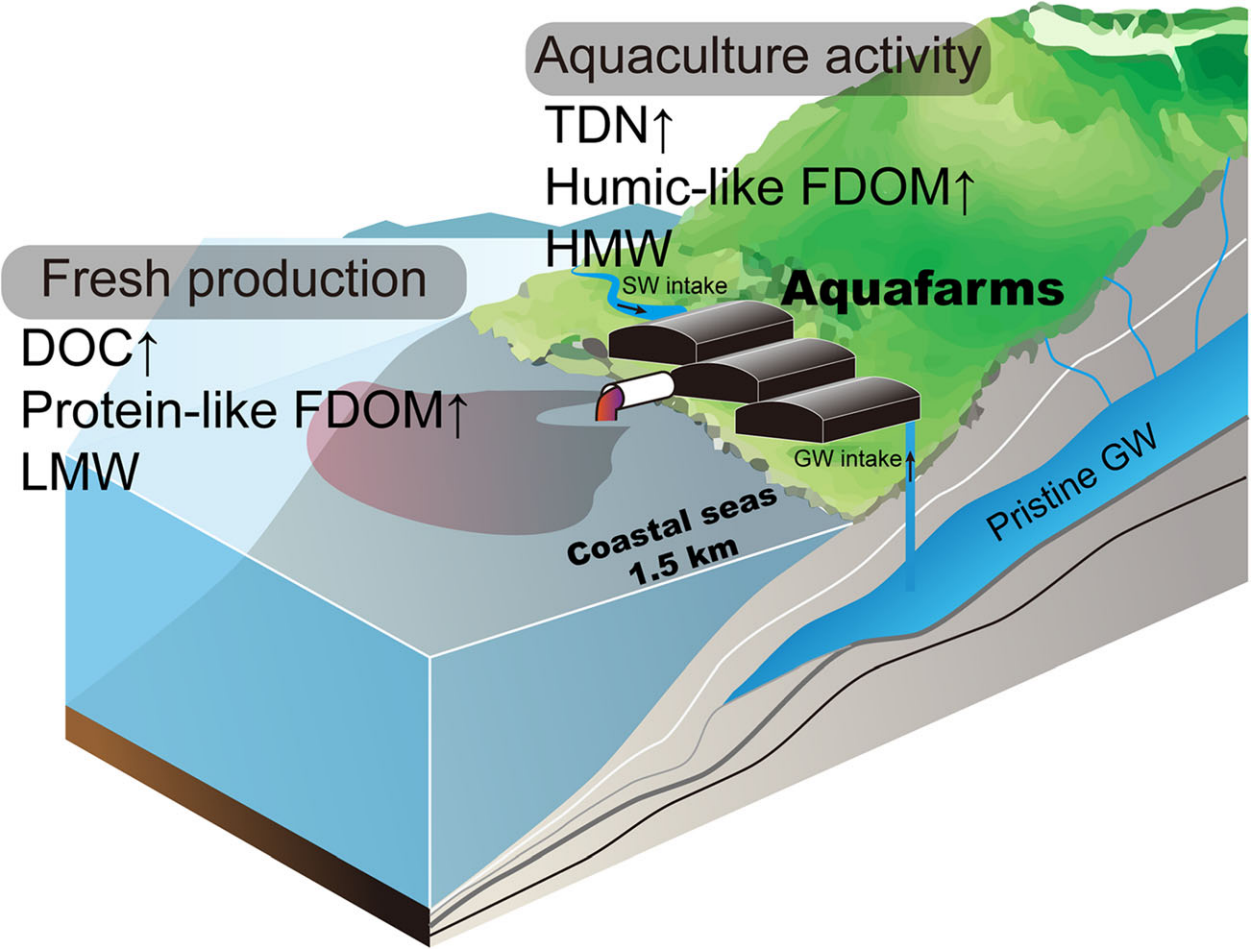
A schematic diagram illustrating the properties and origins of DOM near the coastal Jeju Island affected by the discharge from the aquaculture facilities

The unclear spatial distributions of DOC and TDN can be attributed to random aquaculture activities depending on the life cycle of cultured fish and environmental responses, such as hyperthermal and hypoxic events. In addition, the mixing of DOM originating from various sources and production mechanisms results in inconclusive characteristics of the distribution of DOM in this region. DOM pools contain chromophores and fluorophores with unique optical properties related to their origin and behaviour (Stedmon and Nelson 2015). The groupings of all PARAFAC components and absorbance parameters using PCA analysis identified the common behaviours of DOM. Based on the PCA results, the first group clustered along PC1 showed positive loadings of TDN, *a*_*350*_, *SUVA*_*254*_, C1, and C3, but negative loadings of *S*_*250–600*_ (Fig. 4), indicating that aquaculture activity was the primary source of the coastal DOM (Fig. 6). In addition, the positive values of the PCA scores clustered along PC1 for most drainage samples (avg. 1.99 ± 2.57), and the exponential increase of TDN and the humic-like components close to the outlet were also supported (Fig. 5). Humic-like components were previously found to accumulate in the culture water derived from fish faeces, uneaten feed, and fish blood (Leonard et al. 2002; Nimptsch et al. 2015; Yamin et al. 2017), which is consistently with our results. In particular, although the organic substances released from fish feed and faeces can increase microbial activity, the leaching of the dissolved organic nitrogen fraction results in an increased concentration in the culture water (Aguilar-Alarcón et al. 2020; Burford and Williams 2001).

Furthermore, *a*_*350*_ and *SUVA*_*254*_ provide a measure of the optical intensity of DOM and are significantly associated with molecular weight and aromaticity (Chin et al. 1994). *S*_*250–600*_ typically exhibits an inverse relationship with the molecular weight and is regarded as a proxy for the molecular weight and aromaticity of DOM (Blough and Del Vecchio 2002; Helms et al. 2008; Weishaar et al. 2003). The cluster of *a*_*350*_ and *SUVA*_*254*_ together with TDN and the humic-like components and the negative loading of *S*_*250–600*_ suggest that the aquaculture-driven DOM contains light-sensitive organic substances characterised as humified DOM with higher molecular weight and aromaticity. The humified DOM may be attributed to the microbial activity in the culture water (Chaves et al. 2021).

DOC and C2 clustered along PC2 (Fig. 4), indicating that both parameters were closely related and had a common source. This was consistent with a previous finding that indicated that a significant increase in DOC concentrations was mainly associated with an increase in protein-like DOM (Nimptsch et al. 2015). Based on the results from the PARAFAC model and the OpenFluor database, C2 was produced by the fresh biological activity in the marine environment (Hambly et al. 2015, Osburn et al. 2016, Shutova et al. 2014; Table 1). The distributions of DOC and C2 in the coastal region demonstrated significant scattering and increased with distance, with the highest values observed within a 500–1000 m distance from the aquafarm (Fig. 5). We speculated that the DOC and C2 in the coastal region were not directly derived from the aquafarms. Typically, coastal aquafarms largely contribute to the nutrient budget, often causing eutrophication (Wang et al. 2012). The input of nutrients enables the stimulation of fresh biological production, eventually increasing in the protein-like FDOM. Recently, Chaves et al. (2021) demonstrated a spectral shift from the humic-like FDOM components towards protein-like FDOM components with distance from the aquafarms due to fish farming. Hence, the results of PCA and the distribution patterns indicated that the DOC was freshly produced by the biological activity near the aquafarms, likely due to the nutrient input from wastewater (Figures 4–6). This protein-like FDOM was dominant at lower molecular weights (Cuss and Guéguen 2015; Kowalczuk et al. 2009). The increase in *S*_*250–600*_ within 500 m suggested that the low-molecular-weights DOM seemed to be produced *in situ* near the aquafarms (Figure 5). In addition, the notable feature was the lowest *SUVA*_*254*_ value near 500 m distance, indicating the low aromaticity DOM.

Based on the relatively lower variable contributions of C4 and C5 (Fig. 4), these components were weakly associated with the other parameters, and showed extremely limited matches with respect to the OpenFluor database (Table 1). Because the fluorescent peak of C4 was similar to that of the marine humic-like component (M peak) defined by Coble (1996), C4 originated from in situ production, followed by microbial metabolism. However, significantly low concentrations of C4 suggested a minor contribution of DOM (Fig. 5). The peak of C5 was similar to that of tryptophan amino acid-like fluorescence (Table 1). However, the distribution pattern of C5 with a distance from the outlet resembled that of the humic-like FDOM components (Fig. 5). In previous studies, some FDOM components similar to C5 have been observed in the effluent of aquafarms (Fellman et al. 2010; Nimptsch et al. 2015), indicating that C5 is a unique fluorescence signal derived from the aquafarm. However, this component was previously reported to be less affected by aquafarms than other components (Nimptsch et al. 2015). Thus, we concluded that C4 and C5 may be rare FDOM components in natural water.

## Conclusions

The use of a mixture of coastal groundwater and offshore water in the aquafarms lowered the pH and salinity in the drainage and coastal regions near the discharge outlet. The elevated DOC concentration in the drainage in comparison with that in natural groundwater was attributed to the aquaculture activity and intake of offshore water. However, the distribution of DOC in the coastal region demonstrated significant scattering and inconclusive distribution patterns. The TDN concentration exponentially increased close to the discharge outlet, and this enrichment might be derived from fish faeces, uneaten feed, and fish blood. In summary, the relatively higher concentrations of DOC and TDN in the wastewater are affected by aquaculture activity. Based on the results of PCA, the primary factor (PC1; 48.1%) controlling the distribution of organic matter in this region is aquaculture activity, whereas the secondary factor (PC2; 18.8%) is coastal autochthonous production in the water column. The simultaneous positive loadings of TDN with *a*_*350*_, *SUVA*_*254*_, and humic-like components, as well as the negative loadings of *S*_*250–600*_, suggest that the aquaculture-driven DOM contains enriched TDN with light-sensitive organic substances characterised as humified DOM with higher molecular weight. The cluster of DOC and protein-like components along PC2 implys that DOC is mainly produced by fresh biological activity. In addition, with an increase in *S*_*250–600*_ within 500 m, the DOC seems to contain low-molecular-weight substances. Based on the results of this study, the optical properties of organic matter can be used as tracers to determine quantitative and qualitative information on organic pollution from aquafarms. However, because the PCA elucidates merely less than 70% of the variables, further studies are necessary to identify other processes that can contribute to the organic matter distribution in Jeju Island, particularly groundwater-driven organic matter. Furthermore, the connection between aquaculture-driven wastewater and coastal green tides is necessary to understand the application of isotopic approaches such as δ^13^C and δ^15^N.

## Declarations

### Availability of data and materials

The datasets generated during and/or analysed during the current study are available from the corresponding author on reasonable request.
